# 

**Published:** 1910-03

**Authors:** 


					﻿Sixty =Ttfth Year.
Vol. LXV. MARCH, 1910.	No. 8
BUFFALO
MEDICALJOURNAL
Established 1845 by AUSTIN FLINT M. D.
EDITOR:
WILLIAM WARREN POTTER, »[. D.
ASSISTANT EDITORS:
NELSON W. WILSON, M. D.	JAMES WRIGHT PUTN4M,.M-IX-
ASSOCIATE EDITORS:
ERNEST WENDE, M. D. JOHN A. RAFTER, M. D.
JOHN PARMENTER, M. D.	JOHN A. MILLER, Ph. D.
MAUD J. FRYE, M. D.
				

